# Clinicopathological Profile of Prostate Cancer Patients at a Tertiary Hospital in the Eastern Cape, South Africa

**DOI:** 10.3390/ijerph23060744

**Published:** 2026-06-02

**Authors:** Monde Magadla, Mojisola Clara Hosu, Linda Sobekwa, Mirabel Kah-Keh Nanjoh

**Affiliations:** 1 School of Public Health, Faculty of Medicine and Health Sciences, Walter Sisulu University, Mthatha 5117, South Africa; mmagadla@aol.com (M.M.); lsobekwa@wsu.ac.za (L.S.); 2 School of Pathology, Faculty of Medicine and Health Sciences, Walter Sisulu University, Mthatha 5117, South Africa; mhosu@wsu.ac.za

**Keywords:** prostate cancer, clinicopathological profiles, prostate-specific antigen, Gleason score, TNM staging, risk stratification

## Abstract

**Highlights:**

**Public health relevance—How does this work relate to a public health issue?**

- Prostate cancer is one of the leading cancers among men in South Africa. Describing who is affected, how they present, and at what stage provides essential evidence to guide cancer control planning and prioritization at provincial and national levels.
- Understanding patterns of late presentation, high PSA levels, and advanced disease supports evidence-based discussions on targeted screening approaches for high-risk groups, rather than one-size-fits-all screening policies.

**Public health significance—Why is this work of significance to public health?**

- The study highlights its ability to inform population-level cancer control, health equity, and health-system planning in a high-burden setting.
- The study aims to generate locally relevant evidence essential for strengthening prostate cancer surveillance, guiding public health interventions, and improving outcomes in South Africa’s Eastern Cape Province.

**Public health implications—What are the key implications or messages for practitioners, policy makers and/or researchers in public health?**

- For practitioners, the high proportion of symptomatic and advanced prostate cancer at diagnosis underscores the need for earlier risk recognition in primary health care, particularly among men aged ≥50 years and those with comorbidities or family history.
- Policy support for men’s health programs, community awareness initiatives, and task-shifting (e.g., training nurses and community health workers) can help reduce delays in presentation and diagnosis.

**Abstract:**

Background: Prostate cancer remains a major cause of cancer-related morbidity and mortality among men worldwide. Limited access to oncology services contributes to late presentation, delayed diagnosis, and treatment. This study describes the clinicopathological profile of prostate cancer and identifies factors associated with disease severity at presentation in an Eastern Cape population. Methods: A retrospective cross-sectional study was conducted among men diagnosed with prostate cancer at a tertiary hospital between 2014 and 2024. Demographic, clinical, and pathological data were extracted. Descriptive analyses were performed, and multivariable logistic regression was used to identify independent predictors of advanced disease. Results: The study included 202 patients with a mean age of 67.2 years. Thirty-four (16.8%) reported a family history of prostate cancer, and 62.4% had never undergone PSA screening before diagnosis. Elevated PSA levels were common (60.4%), and more than half of patients presented with advanced disease (54.5%). High-risk and very high-risk disease were identified in 44.1% and 21.3% of patients, respectively. Lack of prior PSA screening was independently associated with high-risk disease (aOR 2.4, 95% CI 1.1–5.0), advanced stage at presentation (aOR 2.4, 95% CI 1.2–4.8), and PSA > 20 ng/mL. Conclusions: There is a high burden of late-stage, high-risk prostate cancer at presentation. These findings highlight ongoing challenges in early detection and emphasize the need for improved awareness, screening, and referral pathways to improve outcomes.

## 1. Introduction

Prostate cancer is a major global health concern, ranking among the most common cancers in men and a leading cause of cancer-related morbidity and mortality worldwide [1,2,3]. In 2022, an estimated 1.47 million new cases were reported globally, making prostate cancer the second most frequently diagnosed cancer in men and the fifth leading cause of cancer mortality [4,5]. Although prostate cancer incidence is highest in high-income countries, largely due to widespread screening, mortality rates are disproportionately higher in low- and middle-income countries (LMIC), reflecting late diagnosis, limited access to care, and constrained health-system capacity [6]. In Africa, prostate cancer is a growing public health concern. Men of African ancestry are known to have a higher risk of developing aggressive disease and poorer outcomes compared with other populations [7,8,9,10]. In sub-Saharan Africa, prostate cancer is often diagnosed at advanced stages, with high prostate-specific antigen (PSA) levels, high Gleason scores, and metastatic disease at presentation [11]. These patterns are influenced by limited awareness, low screening uptake, sociocultural barriers, and health-system challenges that delay diagnosis and referral.

South Africa mirrors these regional trends, with prostate cancer among the most commonly diagnosed cancers in men, with an incidence of 68/100,000 [12,13]. Although PSA testing is available, routine population-based screening is not widely implemented, and many patients present through symptomatic or incidental detection rather than structured early-detection pathways [14]. Marked disparities persist across provinces, particularly in historically underserved regions such as the Eastern Cape, where poverty, rural residence, and limited specialist services may contribute to delayed presentation and advanced disease.

Despite the substantial burden, published data on the clinicopathological profile of prostate cancer patients at presentation in the Eastern Cape Province remain limited. Understanding age distribution, racial representation, screening history, PSA levels, Gleason scores, tumor–node–metastasis (TNM) staging, and metastatic patterns at diagnosis is essential for informing public health planning, strengthening early-detection strategies, and guiding equitable cancer care. This study, therefore, aims to describe the clinicopathological characteristics of prostate cancer patients managed at a tertiary hospital in the Eastern Cape, providing context-specific evidence on disease presentation in a high-burden, resource-limited setting. Such data are critical for supporting targeted interventions and improving prostate cancer outcomes at the provincial and national levels.

## 2. Materials and Methods

### 2.1. Study Design and Setting

This study used a cross-sectional design with a retrospective chart review and baseline data collected at diagnosis. The cross-sectional period spanned January 2014 to December 2024, during which routinely collected clinical data were analyzed to address the study objectives. The study was conducted at Frere Hospital, the second-largest tertiary hospital in the Eastern Cape Province, South Africa. Frere Hospital is located within the Buffalo City Metropolitan Municipality on the east coast of the Eastern Cape. The municipality includes the urban centers of East London, Bisho, and King William’s Town, the large townships of Mdantsane and Zwelitsha, and surrounding rural communities [15]. Frere Hospital serves as a major oncology referral center in the province. Its 67-bed oncology unit provides specialized, state-of-the-art cancer care and manages more than 70% of cancer patients in the Eastern Cape. According to the hospital’s 2024 database, the unit receives over 2000 new oncology patients annually, underscoring its central role in cancer diagnosis and management within the province.

### 2.2. Study Population

All medical records of patients with pathologically diagnosed prostate cancer treated at Frere Hospital’s oncology section during the study period made up the target group for this investigation.

### 2.3. Sampling Method

A probabilistic simple random sampling technique was used to select the study population. Each eligible patient file was assigned a unique coded identifier, and a complete sampling frame of the study population was compiled. Random selection was then performed using EPIDAT software for epidemiological analysis of tabular data (v 3.1) to generate the required number of random identifiers corresponding to the target sample size.

### 2.4. Sample Size Calculation

The sample size was calculated using the following formula:

Z^2^ p(1 − p)/e^2^.

Using a prevalence of 21.3% reported in a previous study [12], a 5% margin of error, and a 95% confidence interval, the minimum required sample size was 258 patients. To account for potential missing or incomplete data, an additional 10% (n = 26) was added, yielding an anticipated sample size of 284 records. However, due to limited access to records during the data collection period, the final sample comprised 202 medical records from patients who met the inclusion criteria.

### 2.5. Inclusion and Exclusion Criteria

The study included adult male patients aged 40 years or older who received initial care and follow-up at the study facility during the 10-year study period. Eligible patients were residents of East London, the townships of the Buffalo City Metropolitan Municipality, the Amathole, OR Tambo, Chris Hani, and Joe Gqabi Health Districts, and the surrounding rural districts of the Eastern Cape Province. Patients were either self-referred or referred from other districts or healthcare facilities for cancer treatment services.

Patients were excluded if they changed their residential address during the study period, had received their initial diagnosis in another province and attended the study facility only for follow-up, were enrolled in interventional drug studies or clinical trials, or had incomplete or missing clinical records.

### 2.6. Data Collection

Data were extracted from patient records using a self-designed data collection tool. Collected information included sociodemographic variables such as age, marital status, place of residence, ethnicity, and level of education. Clinical variables captured the presence of typical and atypical prostate cancer symptoms, allowing classification of participants as symptomatic (with typical and/or atypical symptoms) or asymptomatic at the time of hospital contact. Data on presenting comorbidities were collected and used to compute the Charlson Comorbidity Index (CCI) and estimate 10-year survival. The CCI was calculated using the MDCalc online Charlson Comorbidity Index calculator <Charlson Comorbidity Index (CCI)>. Documented comorbid conditions for each patient at the time of diagnosis were entered into the calculator, which generated a total CCI score and a corresponding estimated 10-year survival probability. For descriptive and analytical purposes, estimated 10-year survival probabilities were categorized as <50%, 50–90%, and >90%. This estimated survival probability is a prognostic measure derived from comorbidity burden rather than observed survival.

Pathological characteristics were extracted from medical records as documented by consulting physicians and urologists. In accordance with South African urological practice at the study site, pathological assessment followed standard diagnostic procedures, including screening, digital rectal examination, biopsy, and laboratory analyses [16,17]. Variables of interest included PSA levels; disease involvement, documented as localized (in situ), invasive (extension and growth in surrounding prostatic tissues, adjacent organs, or regional lymph nodes), or metastatic (presence or absence of distant spread); Gleason score; tumor stage (T1a–T4); and lymph node involvement (absent, present, or unspecified). In addition, specific pathological features were recorded, including sites of invasion such as lymphovascular invasion and perineural invasion, defined as the presence of malignant cells tracking along or surrounding a nerve fiber [18]. Documented sites of metastasis, such as the bone, cervical spine, and femur, were also recorded. Pathological data were subsequently classified according to the World Health Organization (WHO) Gleason Score Grade Groups, TNM clinical staging (Stage I–IV), the reflected clinicopathological stage (localized Stage I and II, locally advanced Stage III, and advanced Stage IV), and the NCCN risk stratification classification (low, intermediate, high and very high) [19]. Data collection was done by the principal investigator, with support from a trained research assistant, ensuring consistency and accuracy throughout the data extraction process.

PSA screening uptake prior to diagnosis was defined as having undergone at least one formal PSA test before prostate cancer diagnosis (Yes/No). The diagnostic initiation mechanism was categorized as voluntary (client-initiated) or opportunistic (health provider-initiated), adapted from the South African HIV screening framework [20]. This differs from international PSA opportunistic testing, which is based on PSA testing outside organized population-based screening [21,22,23]. Diagnostic initiation pathways were defined by symptom status and the mechanism of PSA testing initiation.

The primary outcomes of this study were adverse disease characteristics at diagnosis, including high-risk prostate cancer, advanced disease stage, and PSA > 20 ng/mL. Multivariable logistic regression was used to identify factors independently associated with these outcomes.

### 2.7. Validity and Reliability

The study’s internal validity was strengthened through careful design, pilot testing, and review of the data collection instrument to ensure alignment with the study objectives. Before full data collection, the tool was pilot tested with 13 patient files to evaluate the data collection process and confirm that all relevant variables were captured appropriately. Feedback from the pilot informed refinements to the data collection instrument, after which it was finalized for use. The data collection tool was serialized to prevent duplication and was reviewed and approved by the study supervisors before implementation. All patient files were independently cross-checked, and the extracted data were verified for completeness and accuracy before analysis. The patient files used during the pilot phase were included in the final study sample.

### 2.8. Statistical Analysis

Frequencies and percentages were calculated for categorical variables. Comparisons of medians were performed using the Mann–Whitney U test or the Kruskal–Wallis H test, and comparisons of means were performed using the independent-samples *t*-test, as appropriate. Spearman’s rank-order correlation analysis was performed for all pathological markers. A log_10_ transformation was applied to PSA values, and PSA values > 1000 were truncated to 1000 and then log_10_-transformed. Linear regression was performed to assess associations between log_10_-transformed PSA and WHO Grade Group and TNM clinical stage. Collinearity diagnostics showed no evidence of multicollinearity (tolerance values were 0.796, and VIFs were 1.257). Associations between categorical variables were assessed using Pearson’s chi-square (χ^2^) test. For ordinal variables with three or more ordered categories, the χ^2^ test for trend (linear-by-linear association) was used. Fisher’s exact test (for 2 × 2 tables) or Monte Carlo–estimated exact χ^2^ tests (for larger tables) were applied when more than 25% of expected cell counts were less than five. When an overall Pearson’s χ^2^ or Kruskal–Wallis test was statistically significant, post hoc pairwise comparisons were performed with Bonferroni correction. Multivariable analysis was conducted to identify independent predictors of study outcomes [high-risk prostate cancer (NCCN high-risk and very high-risk), advanced disease stage (TNM stage IV), and PSA > 20 ng/mL]. A *p*-value < 0.05 was considered statistically significant. All statistical analyses were performed using IBM SPSS Statistics for Windows, version 30.0 (IBM Corp., Armonk, NY, USA). Results were summarized in tables and illustrated with bar graphs or error bars, as appropriate.

## 3. Results

### 3.1. Sociodemographic and Screening Characteristics of the Study Population

The majority (71.8%, n = 145) were married, 6.9% (n = 14) were single, and 27 (13.4%) had unknown status. The majority of participants (73.4%) were retirees, while a few were unemployed (8.4%), employed (6.9%), or had unknown status (9.4%). More than three-quarters (87.1%) were black. The mean age at baseline diagnosis of prostate cancer in the study population was 67.2 years, with a minimum of 42 years, a maximum of 88 years, and a standard deviation of 7.9 years. Most patients were in the 60–69-year age group (46.5%). Furthermore, more than half (54.5%) resided in urban areas, while 28.7% and 16.8% resided in rural and peri-urban areas, respectively. The precise municipal demarcation of these residences, in descending order of representation, was Buffalo City (59.4%), Amathole (20.3%), Chris Hani (11.4%), O.R. Tambo (5.9%), Joe Gqabi (2%), and Alfred Nzo (1%) (Table 1).

The screening rate was very low at 37.6%, and the majority (62.4%) had never been screened for PSA before diagnosis. Opportunistic testing leading to diagnosis, mainly driven by typical and atypical prostate cancer symptoms, was observed in most participants (63.4%). Family predisposition was noted in 34 (16.8%) participants. The median number of years spent at the study site receiving cancer care services was two years. The majority (55%) received cancer care services for 1–5 years, followed by those who received them for less than 1 year (22.8%).

### 3.2. Clinical Profile

**Figure 1:**
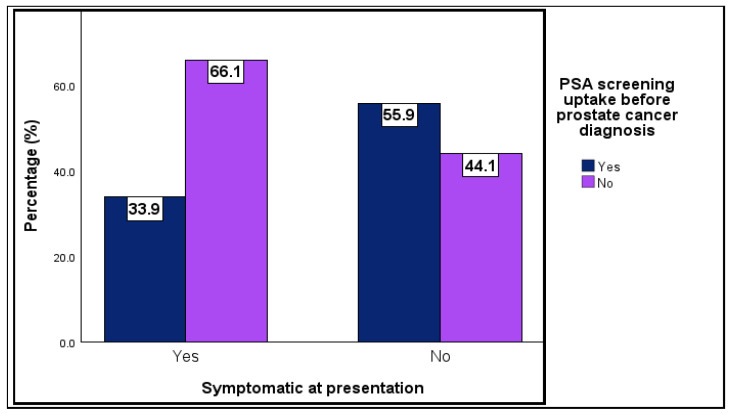
Distribution of PSA screening history by symptomatic status at presentation among prostate cancer patients.

**Figure 2:**
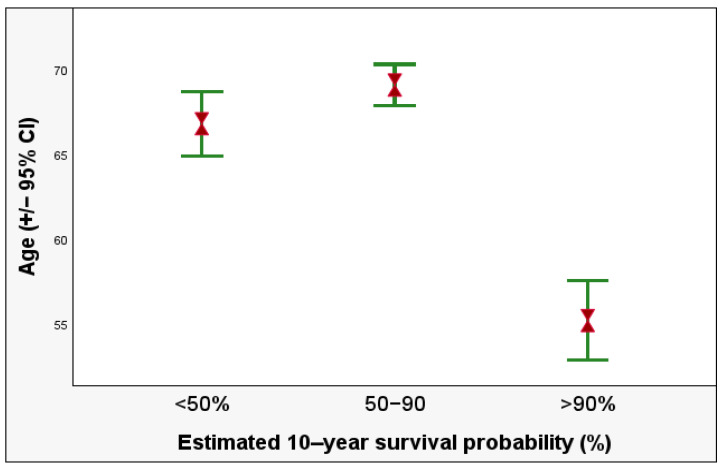
Comparison of patients’ age by CCI-Estimated 10-Year Survival Groups (Error bars represent standard deviation).

The clinical features reported in this section include presentation at diagnosis, comorbidity, and management options. Regarding clinical presentation, the majority (82.2%) were symptomatic at presentation, while the remaining 17.8% were asymptomatic. Participants without prior PSA screening before diagnosis were more likely to be symptomatic at presentation (RR = 1.5 (95% CI: 1.0–2.2; *p* = 0.024)) (Figure 1). Symptomatic participants experienced 1 to 6 of the 24 symptoms, with the three most common being body, back, or suprapubic pain (26.5%); lower urinary tract symptoms (21.1%); and lower-limb weakness (20.5%). The management distribution shows a predominance of external beam radiotherapy (55.0%), followed by androgen deprivation therapy (ADT) (21.3%), with smaller proportions undergoing orchiectomy (9.4%) or managed with active surveillance (10.4%) (Table 2). Likewise, the majority (64.4%) had other morbid conditions; hypertension (88.5%), diabetes mellitus (35.4%), and HIV (11.5%) were the top three of the 11 comorbidities, with a maximum of four comorbidities observed in some cases. Based on the comorbidity data, the Charlson comorbidity index (CCI) was calculated, with a median of 3.5 (interquartile range: 2.0–8.0) and minimum and maximum values of 1 and 15, respectively. Based on the Charlson comorbidity index, the estimated 10-year survival probability at the time of diagnosis was 50–90% for the majority (55.9%), <50% for 36.6%, and >90% for 7.4%. Patients in the lowest predicted survival category (<50%) were, on average, older at diagnosis (mean age = 66.8 ± 8.2 years) than those in the 50–90% group (mean age = 69.1 ± 6.5 years) and the >90% group (mean age = 55.2 ± 4.2 years, *p* < 0.001) (Figure 2). Less frequent presenting symptoms and comorbidities are summarized in Supplementary Table S1.

### 3.3. Pathological Profile

The majority of patients presented with localized disease, indicating that 45.5% of patients’ cancers were still confined to the prostate. Metastasis was identified in 76 patients, of whom 22 had concomitant invasive disease, while 34 patients had invasion without metastasis. The majority of cases with invasive forms had perineural invasion (85.7%, n = 48/56), with two invasive sites in one patient. The site of metastasis was bone (85.5%), with the lumbar bone (60.0%) being the most common. For the single scores, the median Gleason score was 7 (interquartile range [IQR]: 6–8), with a range of 2 to 10 (Table 3).

The median baseline Gleason grading score was 3.4 (IQR: 3.3–4.4), with a minimum of 1.1 and a maximum of 5.5 for double scores. The Gleason scores classified the majority as Group 1 prostate cancer (37.6%). The median PSA level was 26.7 ng/mL (interquartile range: 11.7–193.5), with a range of 1.9 to 21,051.3. After truncation, the median PSA remained unchanged (median truncated PSA = 26.7 ng/mL, IQR 11.7–193.5). The mean (SD) log_10_-transformed PSA was 1.8 (0.9), whereas the mean (SD) log_10_-transformed truncated PSA was 1.7 (0.8). Moreover, the majority of tumors were staged as T1a–T2a (57.6%). In 56 (27.7%), the cancer had spread to one to three nearby lymph nodes, and in 72 (35.6%), it had spread to other parts of the body. Pathologically, the majority (54.5%) had Stage IV or advanced disease, and 89 (44.1%) were confirmed to have high-risk prostate cancer at the time of diagnosis.

#### 3.3.1. Comparison and Correlation Analyses Between PSA and Other Pathological Markers

The median PSA was significantly higher in participants with WHO Grade Group 5 (*p* < 0.001), clinical Stage IV (*p* < 0.001), advanced disease (*p* < 0.001), and high-risk disease (*p* < 0.001). The median PSA varied across age categories but did not reach statistical significance (*p* = 0.154) (Table 4). Pairwise comparisons showed significantly higher PSA levels in WHO Grade Groups 4 and 5 than in Grade Group 1 (*p* < 0.05). Grade Group 5 also had significantly higher PSA levels than Grade Groups 2 and 3 (*p* < 0.05). Additionally, PSA levels were significantly higher in Stage IV disease than in Stage I (*p* < 0.05). Moreover, PSA levels were significantly higher in advanced disease than in localized disease (*p* < 0.05). PSA levels were significantly higher in the high- and very-high-risk groups than in the low- and intermediate-risk groups, whereas no significant difference was observed between the low- and intermediate-risk groups.

Additionally, Spearman’s rank-order correlation analysis was conducted to assess associations between PSA and other prostate cancer pathological markers (Table 5). PSA showed a strong positive correlation with risk-based stage (rho = 0.838, *p* < 0.001), as expected given that PSA forms part of the NCCN risk stratification. A strong positive correlation was observed with tumor stage (rho = 0.721, *p* < 0.001) and a moderate positive correlation with WHO Grade Group (rho = 0.472, *p* < 0.001). Significant positive correlations were also observed between PSA and distant metastatic status (rho = 0.583, *p* < 0.001), clinical stage (rho = 0.596, *p* < 0.001), and overall clinicopathological stage (rho = 0.582, *p* < 0.001). The association between PSA and lymph node metastasis was weak and not statistically significant (rho = −0.074, *p* = 0.298).

Furthermore, linear regression was performed to assess the extent to which tumor grade and clinical stage could predict PSA levels (Table 6). A significant regression was found (*p* < 0.001), and the equation generated from the linear regression was Log_10_ (PSA) = 0.576 + 0.173 (WHO Grade Group) + 0.273 (Clinical stage). Each increase in the WHO Grade Group category was associated with an average 0.173-unit (49%) increase in log_10_-transformed PSA. Similarly, each progression to a higher clinical stage was associated with an average 0.273-unit (88%) increase in log_10_-transformed PSA. Higher WHO Grade Group and clinical stage were significantly associated with higher PSA levels (*p* < 0.001).

#### 3.3.2. Association Between Perineural Invasion and Clinicopathological Markers

Perineural invasion was significantly associated with advanced disease (Stage IV in the TNM classification) (*p* < 0.001), WHO Grade Group 5 (*p* = 0.012), higher PSA levels (*p* = 0.039), and high-risk disease (NCCN risks group) (*p* = 0.023). Post hoc pairwise comparisons were Bonferroni-adjusted. Clinical stage differences were significant (α = 0.05/6 = 0.008) for stage I vs. IV (*p* < 0.001) and stage II vs. IV (*p* = 0.002). Clinicopathological stage differed (α = 0.05/3 = 0.017) only between localized and advanced disease (*p* < 0.001). All other pairs were not significant (*p* > 0.05). No pairwise differences between risk-based groups remained significant after Bonferroni correction (*p* > 0.008) (Table 7).

No significant association was observed between perineural invasion and distant metastasis (*p* = 0.623). In multivariable logistic regression, none of these pathological markers were independently associated with perineural invasion.

#### 3.3.3. Comparison of Baseline Characteristics by Race

Symptomatic presentation was most common among Black patients (85.2%) and least common among White patients (55.0%, *p* = 0.005). A PSA level > 20 ng/mL was significantly more prevalent among Black patients (64.2%, *p* = 0.014) than among Mixed-race (33.3%) and White patients (35.0%) (Table 8). These variables and practices were not independently associated with race in the multinomial logistic regression analysis. Detailed disease classification variables with sparse racial subgroup counts are presented in Supplementary Table S2.

#### 3.3.4. Comparison of Baseline Characteristics by Marital Status

No statistically significant associations were observed between marital status and age group at diagnosis (*p* = 0.114), duration of encounter with the healthcare facility (*p* = 0.216), diagnostic initiation pathway (*p* = 0.415), symptomatic status at presentation (*p* = 0.423), WHO Grade Group (*p* = 0.904), PSA categories (*p* = 0.217), clinical staging (*p* = 0.392), clinicopathological stage (*p* = 0.463), or risk stratification (*p* = 0.998) (Supplementary Table S3).

### 3.4. Independent Predictors of High PSA, Advanced, and High-Risk Prostate Cancer

In the multivariable logistic regression analysis, lack of PSA screening uptake before diagnosis was associated with higher odds of high-risk prostate cancer (adjusted odds ratio [aOR] 2.4, 95% CI 1.1–5.0; *p* = 0.024), advanced disease at presentation (aOR 2.4, 95% CI 1.2–4.8; *p* = 0.015), and PSA > 20 ng/mL (aOR 2.4, 95% CI 1.1–5.0; *p* = 0.020). Symptomatic presentation at diagnosis was also strongly associated with high-risk disease (aOR 3.6, 95% CI 1.4–9.4; *p* = 0.008) and high PSA (aOR 2.7, 95% CI 1.0–7.2; *p* = 0.040). Absence of comorbidity was independently associated with PSA > 20 ng/mL (aOR 2.4, 95% CI 1.1–5.2; *p* = 0.030) (Table 9). Other socio-demographic and clinical covariates with no statistically significant associations across outcomes are presented in Supplementary Table S4.

## 4. Discussion

Our findings provide essential baseline data for developing targeted interventions to address the prostate cancer burden in South Africa and could inform efforts in other sub-Saharan African countries.

An important factor influencing poor quality of life among prostate cancer survivors is age at diagnosis [14]. Prostate cancer primarily affects older men, with about 60% of cases diagnosed at age 65 or older, and it is rare in men under 40 [24]. Similarly, studies of South African and Nigerian men reported a mean age at diagnosis of 66 years [25]. Our findings are consistent with a South African hospital-based study, which reported a central age distribution in the mid-60s [14]. However, registry data from 10 sub-Saharan African countries indicate a slightly higher mean age of about 70 years [11], suggesting that our cohort is comparable yet somewhat younger. This may reflect differences in access, referral pathways, and diagnostic practices. The advanced age at diagnosis likely indicates delayed presentation, as early-stage prostate cancer is often asymptomatic in younger men.

The high proportion of married men in this study (71.8%) is comparable to findings from the Free State, where approximately 75% were married [14]. This is clinically relevant because household support may influence health-seeking behavior, timely presentation, and continued engagement with treatment and follow-up. However, no statistically significant associations were observed between marital status and age at diagnosis, duration of healthcare contact, diagnostic pathway, symptomatic presentation, WHO Grade Group, PSA category, clinical staging, or risk stratification.

The predominance of Black patients (87.1%) reflects the patient profile of major public oncology services in the Eastern Cape and is consistent with routine data from Frere Hospital [26]. Descriptively, Mixed-race patients had longer engagement with healthcare services, whereas Black patients were more often diagnosed after symptom-driven testing and were more likely to be symptomatic at diagnosis. White patients were more frequently diagnosed through asymptomatic screening. Black patients also had higher PSA levels (>20 ng/mL) and were more often classified as very high risk, whereas Mixed-race and White patients were more represented in intermediate- and high-risk groups. These patterns were not independently associated with race in multinomial logistic regression, suggesting that other factors, such as healthcare access, health-seeking behavior, or disease presentation, may influence them.

The predominance of urban residents highlights inequities in prostate cancer screening among rural populations, a finding also reported internationally [27]. Given that the study sites are urban, the high proportion of patients living within municipal boundaries likely reflects accessibility. Similar studies in Southern Africa report lower screening uptake and more advanced presentations among rural men, with disparities persisting even as testing opportunities expand [28,29,30,31].

The proportion reporting a family history of cancer aligns with South African hospital-based findings, including those from the Free State, where about 15% reported cancer among first-degree relatives [14]. Slightly higher proportions have been reported in primary healthcare settings in the Free State (18.8%) [32], and substantially higher figures in Nigeria (32.0%), alongside poor awareness of family history [33], underscoring variability in awareness and documentation.

Prior formal PSA screening was low, consistent with broader South African data showing limited screening uptake. Only 37.8% of primary care providers report offering PSA screening or health talks [34], and community data from Limpopo indicate extremely low lifetime PSA testing, particularly in rural areas [35]. Most diagnoses in this study arose from opportunistic PSA testing during evaluations for unrelated or nonspecific symptoms, highlighting reliance on incidental rather than systematic detection.

Most patients (82.2%) were symptomatic at presentation, commonly with pain, consistent with studies from South Africa and India that report frequent LUTS and back or bone pain suggestive of advanced disease [8,14,36]. These symptoms are often nonspecific and linked to benign conditions, yet they drive clinical presentation. The proportion of symptomatic patients is higher than in settings with more routine PSA testing. Notably, lower-limb weakness, associated with neurologic complications of advanced disease, was relatively frequent, reflecting delayed presentation [8]. Comorbid conditions were common (64.4%), particularly hypertension (88.5%), diabetes (35.4%), and HIV (11.5%). Hypertension and diabetes are age-related and linked to inflammatory pathways associated with poorer cancer outcomes [37]. Hypertension prevalence exceeded that reported in other populations [38,39], suggesting both genetic and environmental influences. These comorbidity patterns mirror broader South African multimorbidity profiles [40]. HIV adds further clinical complexity, with implications for infection risk, treatment tolerance, drug interactions, and the need for individualized, multidisciplinary care.

Nearly half of patients had localized disease, while 37.6% had metastatic disease and 16.8% had invasive forms, predominantly with perineural invasion (PNI). The prevalence of localized disease was lower than that reported in Ghana [41], whereas metastatic disease was lower than rates reported elsewhere [42,43,44] but with a higher burden of bone metastasis [42,44,45]. Bone was the main metastatic site, consistent with findings from Ghana [41] and in contrast to lymph node–predominant patterns reported in India [46]. Among patients with invasive disease, PNI was common and clinically relevant, as it is associated with aggressive behavior and adverse outcomes, including bone metastasis [47]. The reported prevalence applies specifically to a subgroup of patients with histologically confirmed invasive disease for whom PNI status was documented, rather than to the entire population. PNI was significantly associated with advanced stage, WHO Grade Group 5, and higher PSA levels. While PNI is linked to poorer outcomes, its independent prognostic value remains inconsistent across studies [48].

The findings of this study indicate that most patients presented with advanced, aggressive prostate cancer, as reflected by markedly elevated PSA levels, advanced clinical stage, and high-risk classification. Over 60% had PSA levels greater than 20 ng/mL. According to NCCN guidelines, PSA levels above 20 ng/mL are strongly associated with high-risk disease, while levels exceeding 100 ng/mL suggest extensive tumor burden or metastatic disease [49]. These patterns point to delayed diagnosis and limited access to early screening, consistent with reports from sub-Saharan Africa [50]. Clinical staging showed that 54.5% of patients had advanced disease, indicating presentation after substantial disease progression. Risk stratification further confirmed this trend, with nearly two-thirds categorized as high- or very high-risk groups known to have higher risks of recurrence, metastasis, and prostate cancer-specific mortality [51].

Contemporary evidence highlights the roles of genetic susceptibility, environmental exposures, and population-specific risks, particularly among men of African ancestry, in shaping prostate cancer burden and aggressiveness [52]. In settings where population-wide screening is not feasible, prevention strategies increasingly emphasize risk-adapted and opportunistic PSA testing [53]. Advances such as multiparametric MRI and targeted biopsy have improved detection and risk stratification in well-resourced settings [54], and insights into tumor heterogeneity and androgen receptor signaling further inform understanding of disease progression [55].

The high proportion of patients presenting with advanced and metastatic disease in this study has important therapeutic implications. Evidence supports intensifying androgen deprivation therapy with combination approaches, including androgen receptor pathway inhibitors or chemotherapy, which have improved survival in advanced disease [56,57,58]. Precision oncology, guided by genomic profiling and targeting DNA damage repair pathways, offers additional options for selected patients [59]. Treatments such as PARP inhibitors, immune checkpoint inhibitors, PSMA-targeted radioligand therapy, and AKT inhibitors are now used clinically [60]. However, limited access to these advanced diagnostic and therapeutic modalities in our setting continues to constrain their implementation and underscores persistent disparities in prostate cancer care.

Although the findings are robust, several limitations should be considered. First, as a single-center, tertiary hospital-based study, the results may not be fully generalizable to primary or district-level settings and may overrepresent advanced disease because of referral patterns. Second, the retrospective cross-sectional design relied on routinely recorded data, which may be affected by incomplete documentation, missing information, or misclassification, particularly for family history, symptom duration, and screening history. Third, data on coexisting conditions, such as BPH, were inconsistently captured. Fourth, the CCI reflects all-cause rather than cancer-specific mortality risk, limiting oncologic interpretation, and variability in diagnostic investigations over time may have influenced staging and risk stratification. Furthermore, long-term treatment outcomes and survival were not assessed, limiting inference to disease presentation. Although the study did not reach the initially calculated sample size, it was adequately powered to detect large, clinically meaningful effects. Nonetheless, non-significant findings for the WHO grade group, PSA category, and clinicopathological stage should be interpreted with caution.

### Future Research

Future studies should examine barriers to early presentation, including health-seeking behavior, access to care, and health-system constraints. Longitudinal research linking clinicopathological features at diagnosis with social support structures and their effects on presentation timing, treatment outcomes, and survival would strengthen evidence to improve care pathways. In addition, strengthening population-based surveillance and cancer registries is essential for monitoring trends and evaluating public health interventions.

## 5. Conclusions

This study offers important insights into the clinicopathological profile of prostate cancer patients in a resource-constrained South African setting. The findings highlight a predominance of symptom-driven cases and elevated PSA levels at diagnosis, as well as a substantial burden of advanced-stage and high-risk disease. Key predictors, including PSA screening and symptomatic presentation, were associated with the study outcomes, underscoring the importance of early detection pathways. Overall, the results point to critical gaps in systematic screening and timely diagnosis, with important implications for strengthening early detection strategies, improving healthcare access, and informing context-specific prostate cancer control policies in underserved settings.

## Figures and Tables

**Table 1:** Characteristics of prostate cancer patients managed at Frere Hospital.

Variables	Categories	Count (%); N = 202
Age groups	Less than 50 years	2 (1.0)
	50–59 years	32 (15.9)
	60–69 years	92 (45.5)
	70–79 years	62 (30.7)
	More than 80 years	14 (6.9)
Initial age (years) at diagnosis	Mean ± standard deviation	67.2 ± 7.9
Race	Black	176 (87.1)
	Mixed	6 (3.0)
	White	20 (9.9)
Marital status	Single	14 (6.9)
	Married	145 (71.8)
	Divorced/Separated	3 (1.5)
	Widower	13 (6.4)
	Unknown	27 (13.4)
Employment status	Employed	14 (6.9)
	Unemployed	17 (8.4)
	Self-employed	4 (2.0)
	Retired	148 (73.3)
	Not Stated	19 (9.4)
District municipality of residence	Amathole	41 (20.3)
	Alfred Nzo	2 (1.0)
	Buffalo City Metropolitan	120 (59.4)
	Chris Hani	23 (11.4)
	Joe Gqabi	4 (2.0)
	Oliver Reginald Tambo	12 (5.9)
Place of residence	Rural	58 (28.7)
	Urban	110 (54.5)
	Peri-urban	34 (16.8)
Duration of care	<1 year	46 (22.7)
	1–5 years	111 (55.0)
	>5 years	45 (22.3)
Duration of care (years)	Median (IQR)	2 (1–5)
PSA screening uptake before diagnosis	Yes	76 (37.6)
	No	126 (62.4)
Diagnostics initiation mechanism	Opportunistic testing leading to diagnosis	128 (63.4)
	Voluntary testing leading to diagnosis	74 (36.6)
Diagnostic initiation pathway	Symptoms-driven voluntary testing	55 (27.2)
	Symptoms-driven opportunistic testing	111 (55.0)
	Asymptomatic voluntary testing	19 (9.4)
	Asymptomatic opportunistic testing	17 (8.4)
Family history of cancer	Yes	34 (16.8)
	No	126 (62.4)
	Not known	42 (20.8)

PSA-prostate specific antigen, IQR-interquartile range.

**Table 2:** Baseline clinical features and treatment of prostate cancer patients.

Clinical Features	Yes, n (%)	No, n (%)
Symptomatic at presentation	166 (82.2)	36 (17.8)
Named symptoms at presentation; n = 166		
Body, back, suprapubic pain	44 (26.5)	122 (73.5)
Lower urinary tract symptoms (LUTS)	35 (21.1)	131 (78.9)
Lower limb weakness	34 (20.5)	132 (79.5)
Dysuria	26 (15.7)	140 (84.3)
Urinary retention	23 (13.9)	143 (86.1)
Urinary hesitancy	23 (13.9)	143 (86.1)
Poor stream	20 (12.0)	146 (88.0)
Nocturia	15 (9.0)	151 (91.0)
Urinary obstruction	12 (7.2)	154 (92.8)
Dribbling	10 (6.0)	156 (94.0)
Comorbidity	130 (64.4)	72 (35.6)
Named comorbidity; n = 130		
Hypertension	115 (88.5)	15 (11.5)
Diabetes mellitus	46 (35.4)	84 (64.6)
HIV-infected	15 (11.5)	115 (88.5)
Congestive cardiac failure	10 (7.7)	120 (92.3)
Estimated 10-year survival probability (%)		
<50%	74 (36.6)	
50–90%	113 (55.9)	
>90%	15 (7.4)	
Treatment options		
External beam radiotherapy	111 (55.0)	
Androgen deprivation therapy	43 (21.3)	
Orchiectomy	19 (9.4)	
Other treatment options	7 (3.5)	
Active surveillance	21 (10.4)	
Refused all treatment interventions	1 (0.5)	

**Table 3:** Baseline pathological characteristics of prostate cancer patients.

Variables of Interest	Categories	n (%); N = 202
Disease Involvement	Localised	92 (45.5)
	Metastatic only	54 (26.7)
	Invasive without metastasis	34 (16.8)
	Metastatic and invasive	22 (10.9)
Invasive patterns among patients with invasive disease (n = 56)	Perineural	48 (85.7)
	Diffused	5 (8.9)
	Lymphovascular	2 (3.6)
	Fibromuscular stroma	1 (1.8)
	Lymph nodes	1 (1.8)
Metastatic site; n = 76	Bone	65 (85.5)
	Liver	5 (6.6)
	Bladder	3 (3.9)
	Lungs	2 (2.6)
	Rectum	2 (2.6)
	Unspecified	3 (3.9)
Bone site; n = 65	Lumbar spine	39 (60.0)
	Diffused osteoblastic	12 (18.5)
	Thoracic spine	13 (20.0)
	Pelvic	10 (15.4)
	Cervical spine	2 (3.1)
	Femur	2 (3.1)
	Sacrum	1 (1.5)
	Unspecified	2 (3.1)
WHO Grade Group	Group 1 (GS = 6)	76 (37.6)
	Group 2 (GS = 7 (3 + 4))	32 (15.8)
	Group 3 (GS = 7 (4 + 3))	30 (14.9)
	Group 4 (GS = 8)	29 (14.4)
	Group 5 (GS = 9–10)	35 (17.3)
PSA categories	<10 ng/mL	35 (17.3)
	10–20 ng/mL	45 (22.3)
	>20–100 ng/mL	63 (31.2)
	>100 ng/mL	59 (29.2)
PSA (ng/mL)	Median (interquartile range)	26.7 (11.7–193.5)
Clinical tumour Staging; n = 151	T1a–T2a	87 (57.6)
	T2b–T2c	32 (21.2)
	T3a	14 (9.3)
	T3b–T4	18 (11.9)
Lymph node	NX	109 (54.8)
	N0	37 (18.3)
	N1	56 (27.7)
Metastasis	MX	3 (1.5)
	M0	126 (62.4)
	M1	73 (36.1)
TNM clinical staging	Stage I	78 (38.6)
	Stage II	13 (6.4)
	Stage III	1 (0.5)
	Stage IV	110 (54.5)
Clinicopathological stage	Localized	91 (45.0)
	Locally advanced	1 (0.5)
	Advanced	110 (54.5)
Risk-based stage	Low	20 (9.9)
	Intermediate	50 (24.8)
	High	89 (44.1)
	Very high	43 (21.3)

**Table 4:** Median comparisons of PSA stratified by Age, Clinical stage, Risk-based stage, and Gleason Grade Group in prostate cancer patients.

Variables of Interest	Categories	Median PSA [ng/mL] (IQR)	*p*-Value
Age group	Less than 50 years	121.7 (63.3–180.0)	0.154
	50–59 years	46.0 (10.0–318.2)	
	60–69 years	24.0 (11.4–92.6)	
	70–79 years	25.6 (12.8–362.0)	
	More than 80 years	123.5 (17.4–1995.9)	
WHO Grade Group	Group 1 (GS = 6)	14.9 (10.0–29.4)	<0.001
	Group 2 (GS = 7 (3 + 4))	25.3 (11.6–139.0)	
	Group 3 (GS = 7 (4 + 3))	28.8 (12.7–193.5)	
	Group 4 (GS = 8)	100.0 (21.3–1468.0)	
	Group 5 (GS = 9–10)	376.0 (97.0–1079.0)	
Clinical stage	Stage I	12.9 (9.0–23.2)	<0.001
	Stage II	23.6 (19.6–30.7)	
	Stage III	33.4 (33.4–33.4)	
	Stage IV	111.2 (26.6–1057.0)	
Clinicopathological stage	Localized	13.8 (9.6–24.3)	<0.001
	Locally advanced	33.4 (33.4–33.4)	
	Advanced	111.2 (26.6–1057.0)	
Risk-based stage	Low	7.2 (5.5–9.8)	<0.001
	Intermediate	12.1 (9.6–14.9)	
	High	33.4 (23.8–100.0)	
	Very high	1330.7 (426.5–3792.0)	

**Table 5:** Correlation between PSA and other prostate cancer pathological markers.

		PSA	Gleason Grade Group	Tumour Staging	Lymph Node Metastasis	Distant Metastasis	Clinical Staging	Clinicopathological Stage	Risk-Based Stage
PSA	Rho	1.000							
	*p*-value								
	N	202							
Gleason Grade Group	Rho	0.472 **	1.000						
	*p*-value	<0.001							
	N	202	202						
Tumour Staging	Rho	0.721 **	0.470 **	1.000					
	*p*-value	<0.001	<0.001						
	N	151	151	151					
Lymph node metastasis	Rho	−0.074	0.048	−0.006	1.000				
	*p*-value	0.298	0.500	0.937					
	N	202	202	151	202				
Distant metastasis	Rho	0.583 **	0.411 **	0.566 **	−0.204 **	1.000			
	*p*-value	<0.001	<0.001	<0.001	0.004				
	N	202	202	151	202	202			
Clinical stage	Rho	0.596 **	0.451 **	0.645 **	0.299 **	0.636 **	1.000		
	*p*-value	<0.001	<0.001	<0.001	<0.001	<0.001			
	N	202	202	151	202	202	202		
Clinicopathological stage	Rho	0.582 **	0.468 **	0.558 **	0.295 **	0.650 **	0.978 **	1.000	
	*p*-value	<0.001	<0.001	<0.001	<0.001	<0.001	<0.001		
	N	202	202	151	202	202	202	202	
Risk-based stage	Rho	0.838 **	0.484 **	0.726 **	−0.062	0.641 **	0.636 **	0.603 **	1.000
	*p*-value	<0.001	<0.001	<0.001	0.384	<0.001	<0.001	<0.001	
	N	202	202	151	202	202	202	202	202

** Spearman’s ranked ordered correlation is significant at the 0.01 level (2-tailed).

**Table 6:** Association between WHO Grade Group, clinical stage, and PSA levels.

Pathological Markers of Interest	Unstandardized Coefficient (95% CI)	Standard Error	Standardized Coefficient	*p*-Value
WHO Grade Group	0.173 (0.101–0.245)	0.037	0.293	<0.001
Clinical stage	0.273 (0.196–0.349)	0.039	0.436	<0.001
Constant	0.576 (0.348–0.805)	0.116		<0.001

Dependent variable is Log 10 transformed PSA.

**Table 7:** Clinicopathological markers associated with perineural invasion in prostate cancer at diagnosis.

Variables of Interest	Categories	Perineural Invasion; n (%)		*X*^2^ *p*-Value
		Absent, n = 154	Present, n = 48	
PSA categories	<10 ng/mL	32 (91.4)	3 (8.6)	0.039
	10–20 ng/mL	35 (77.8)	10 (22.2)	
	>20–100 ng/mL	44 (69.8)	19 (30.2)	
	>100 ng/mL	43 (72.9)	16 (27.1)	
WHO Grade Group	Group 1	64 (84.2)	12 (15.8)	0.012
	Group 2	25 (78.1)	7 (21.9)	
	Group 3	20 (66.7)	10 (33.3)	
	Group 4	25 (86.2)	4 (13.8)	
	Group 5	20 (57.1)	15 (42.9)	
Distant metastasis	Absent	98 (77.8)	28 (22.2)	0.623
	Present	56 (73.7)	20 (26.3)	
Clinical stage	Stage I	78 (100.0)	0 (0.0)	<0.001 *
	Stage II	13 (100.0)	0 (0.0)	
	Stage III	1 (100.0)	0 (0.0)	
	Stage IV	62 (56.4)	48 (43.6)	
Clinicopathological stage	Localized	91 (100.0)	0 (0.0)	<0.001 *
	Locally advanced	1 (100.0)	0 (0.0)	
	Advanced	62 (56.4)	48 (43.6)	
Risk-based stage	Low	19 (95.0)	1 (5.0)	0.023 *
	Intermediate	42 (84.0)	8 (16.0)	
	High	60 (67.4)	29 (32.6)	
	Very high	33 (76.7)	10 (23.3)	

* Monte Carlo *p*-value.

**Table 8:** Distribution of demographic, clinical, and pathological attributes by racial groups of prostate cancer patients.

Variable of Interest	Categories	Racial Group			*X*^2^ *p*-Value
		Black, N = 176	Mixed, N = 6	White, N = 20	
		n (%)	n (%)	n (%)	
Age Group	Less than 70 years	108 (61.4)	6 (100.0)	12 (60.0)	0.159 *
	70 years and above	68 (38.6)	0 (0.0)	8 (40.0)	
Duration of encounter with the healthcare facility	≤5 years	139 (79.0)	5 (83.3)	13 (65.0)	0.313 *
	>5 years	37 (21.0)	1 (16.7)	7 (35.0)	
Diagnostic initiation pathway	Symptoms-driven voluntary screening	50 (28.4)	1 (16.7)	4 (20.0)	0.052 ^֎^
	Symptoms-driven opportunistic screening	100 (56.8)	4 (66.7)	7 (35.0)	
	Asymptomatic voluntary screening	13 (7.4)	1 (16.7)	5 (25.0)	
	Asymptomatic opportunistic screening	13 (7.4)	0 (0.0)	4 (20.0)	
Symptomatic at presentation	Yes	150 (85.2)	5 (83.3)	11 (55.0)	0.005 *
	No	26 (14.8)	1 (16.7)	9 (45.0)	
PSA categories	≤20 ng/mL	63 (35.8)	4 (66.7)	13 (65.0)	0.014 *
	>20 ng/mL	113 (64.2)	2 (33.3)	7 (35.0)	

* Fisher’s exact *p*-value, ^֎^ Monte Carlo *p*-value.

**Table 9:** Multivariable logistic regression analysis of factors associated with advanced, high-risk disease, and high PSA at presentation.

Variable	High-Risk Disease		Advanced Disease		High PSA at Screening	
	aOR (95% CI)	*p*-Value	aOR (95% CI)	*p*-Value	aOR (95% CI)	*p*-Value
Age group						
<70 years	1.2 (0.6–2.6)	0.639	0.9 (0.5–1.9)	0.882	1.0 (0.4–2.1)	0.918
≥70 years	Reference	—	Reference	—	Reference	—
Race						
Black	1.5 (0.5–5.2)	0.492	1.1 (0.3–3.4)	0.913	1.7 (0.5–5.9)	0.371
Mixed	0.5 (0.1–4.0)	0.505	0.4 (0.1–3.3)	0.397	0.4 (0.0–3.0)	0.341
White	Reference	—	Reference	—	Reference	—
PSA screening uptake before the diagnosis						
No	2.4 (1.1–5.0)	0.024	2.4 (1.2–4.8)	0.015	2.4 (1.1–5.0)	0.020
Yes	Reference	—	Reference	—	Reference	—
Symptomatic at presentation						
Yes	3.6 (1.4–9.4)	0.008	1.6 (0.6–4.0)	0.317	2.7 (1.0–7.2)	0.040
No	Reference	—	Reference	—	Reference	—
Comorbidity						
No	2.0 (0.9–4.5)	0.075	1.0 (0.5–2.0)	0.949	2.4 (1.1–5.2)	0.030
Yes	Reference	—	Reference	—	Reference	—

## Data Availability

The data presented in this study are available upon reasonable request from the corresponding author.

## Supplementary Materials

The following supporting information can be downloaded at: https://www.mdpi.com/article/10.3390/ijerph23060744/s1. Table S1. Less common presenting symptoms and comorbidities among men diagnosed with prostate cancer (n = 202). Table S2. Disease classification variables with sparse racial subgroup counts in prostate cancer patients. Table S3. Distribution of demographic, clinical, and pathological attributes by marital status of prostate cancer patients. Table S4. Multivariable regression results for socio-demographic and clinical covariates with no statistically significant associations across outcomes.

## Abbreviations

The following abbreviations are used in this manuscript:

aOR	Adjusted Odds Ratio
BPH	Benign prostatic hyperplasia
CCI	Charlson comorbidity index
GGS	Gleason grading score
IQR	Inter quartile range
LMIC	Lower- and middle-income countries
LUTS	Lower urinary tract symptoms
MRI	Magnetic resonance imaging
PNI	Perineural invasion
PSA	Prostate specific antigen
TNM	Tumour-node-metastasis
