# Supplementary material for: Clinicopathological Profile of Prostate Cancer Patients at a Tertiary Hospital in the Eastern Cape, South Africa
Source: Int J Environ Res Public Health. 2026 Jun 2;23(6):744. doi: 10.3390/ijerph23060744 (PMC13299953; doi:10.3390/ijerph23060744)
Supplement: Supplementary file 1 [file ijerph-23-00744-s001.zip › ijerph-4181768-supplementary.pdf]

## Supplementary Tables

**Supplementary Table S1.** Less common presenting symptoms and comorbidities among men diagnosed with prostate cancer (n = 202)

| Clinical features | Yes, n (%) | No, n (%) |
|---|---|---|
| Incontinence | 9 (5.4) | 157 (94.6) |
| Difficulty passing urine | 8 (4.8) | 158 (95.2) |
| Haematuria | 8 (4.8) | 158 (95.2) |
| Burning micturition | 6 (3.6) | 160 (96.4) |
| Bilateral otitis media | 3 (1.8) | 163 (98.2) |
| Erectile dysfunction | 3 (1.8) | 163 (98.2) |
| Enlarged prostate | 3 (1.8) | 163 (98.2) |
| Obstructive uropathy | 3 (1.8) | 163 (98.2) |
| Sexual inadequacy (Painful ejaculation, poor libido, loss of libido) | 3 (1.8) | 163 (98.2) |
| Swelling in the scrotum | 2 (1.2) | 164 (98.8) |
| Painful hip | 2 (1.2) | 164 (98.8) |
| Blood in semen | 1 (0.6) | 165 (99.4) |
| Impaired renal function | 1 (0.6) | 165 (99.4) |
| Paraneoplastic syndrome (cerebellar degeneration) | 1 (0.6) | 165 (99.4) |
| Comorbidity | 130 (64.4) | 72 (35.6) |
| Named comorbidity; n=130 | | |
| Tuberculosis | 6 (4.6) | 124 (95.4) |
| Chronic obstructive airway disease | 5 (3.8) | 125 (96.2) |
| Epilepsy | 5 (3.8) | 125 (96.2) |
| Arthritis | 3 (2.3) | 127 (97.7) |
| Gout | 1 (0.8) | 129 (99.2) |
| Hypercholesterolaemia | 1 (0.8) | 129 (99.2) |
| Parkinsons | 1 (0.8) | 129 (99.2) |

*This supplementary table presents infrequently presenting symptoms and comorbidities (≤5% prevalence) that were excluded from the main table to improve clarity. Percentages are calculated within the total study population unless otherwise specified*.

**Supplementary Table S2.** Disease classification variables with sparse racial subgroup counts in prostate cancer patients

| Variable of interest | Categories | Racial group | | | $X^2$ *p-value* |
|---|---|---|---|---|---|
| | | **Black, N= 176** | **Mixed, N=6** | **White, N=20** | |
| | | **n (%)** | **n (%)** | **n (%)** | |
| WHO Grade Group | Group 1 (GS = 6) | 66 (37.5) | 2 (33.3) | 8 (40.0) | 0.619⊛ |
| | Group 2 (GS =7 (3+4) | 26 (14.8) | 2 (33.3) | 4 (20.0) | |
| | Group 3 (GS =7 (4+3)) | 24 (13.6) | 1 (16.7) | 5 (25.0) | |
| | Group 4 (GS = 8) | 27 (15.3) | 1 (16.7) | 1 (5.0) | |
| | Group 5 (GS = 9-10) | 33 (18.8) | 0 (0.0) | 2 (10.0) | |
| | >20 ng/mL | 113 (64.2) | 2 (33.3) | 7 (35.0) | |
| Clinical staging | Stage I | 67 (38.1) | 4 (66.7) | 7 (35.0) | 0.125⊛ |
| | Stage II | 12 (6.8) | 0 (0.0) | 1 (5.0) | |
| | Stage III | 0 (0.0) | 0 (0.0) | 1 (5.0) | |
| | Stage IV | 97 (55.1) | 2 (33.3) | 11 (55.0) | |
| Clinicopathological stage | Localized | 79 (44.9) | 4 (66.7) | 8 (40.0) | 0.093⊛ |
| | Locally advanced | 0 (0.0) | 0 (0.0) | 1 (5.0) | |
| | Advanced | 97 (55.1) | 2 (33.3) | 11 (55.0) | |
| Risk stratification | Low | 18 (10.2) | 1 (16.7) | 1 (5.0) | 0.076⊛ |
| | Intermediate | 39 (22.2) | 2 (33.3) | 9 (45.0) | |
| | High | 76 (43.2) | 3 (50.0) | 10 (50.0) | |
| | Very high | 43 (24.4) | 0 (0.0) | 0 (0.0) | |

*Fisher's exact p-value, ⊛Monte Carlo p-value

**Supplementary Table S3.** Distribution of demographic, clinical, and pathological attributes by marital status of prostate cancer patients

| Variables of interest | Categories | Marital status | | | $X^2$ *p-value* |
|---|---|---|---|---|---|
| | | **Single N= 14** | **Married N= 145** | **Broken[#] N= 16** | |
| | | **n (%)** | **n (%)** | **n (%)** | |
| Age group | Less than 70 years | 12 (85.7) | 89 (61.4) | 8 (50.0) | 0.114 |
| | 70 years and above | 2 (14.3) | 56 (38.6) | 8 (50.0) | |
| Duration of encounter with the healthcare facility | <1 year | 3 (21.4) | 32 (22.1) | 7 (43.8) | 0.216⊛ |
| | 1-5 years | 9 (64.3) | 75 (51.7) | 8 (50.0) | |
| | >5 years | 2 (14.3) | 38 (26.2) | 1 (6.3) | |
| Diagnostic initiation pathway | Symptoms-driven voluntary screening | 5 (35.7) | 37 (25.5) | 2 (12.5) | 0.415⊛ |
| | Symptoms-driven opportunistic screening | 8 (57.1) | 82 (56.6) | 10 (62.5) | |
| | Asymptomatic voluntary screening | 1 (7.1) | 13 (9.0) | 1 (6.3) | |
| | Asymptomatic opportunistic screening | 0 (0.0) | 13 (9.0) | 3 (18.8) | |
| | Yes | 13 (92.9) | 119 (82.1) | 12 (75.0) | 0.423* |

| | | | | | |
|---|---|---|---|---|---|
| Symptomatic at presentation | No | 1 (7.1) | 26 (17.9) | 4 (25.0) | |
| WHO Grade Group | Group 1 (GS = 6) | 5 (35.7) | 59 (40.7) | 4 (25.0) | 0.904⊛ |
| | Group 2 (GS =7 (3+4) | 3 (21.4) | 21 (14.5) | 3 (18.8) | |
| | Group 3 (GS =7 (4+3)) | 2 (14.3) | 19 (13.1) | 4 (25.0) | |
| | Group 4 (GS = 8) | 2 (14.3) | 24 (16.6) | 1 (6.3) | |
| | Group 5 (GS = 9-10) | 2 (14.3) | 22 (15.2) | 4 (25.0) | |
| PSA categories | ≤20 ng/mL | 7 (50.0) | 53 (36.6) | 9 (56.3) | 0.217 |
| | >20 ng/mL | 7 (50.0) | 92 (63.4) | 7 (43.8) | |
| Clinical staging | Stage I | 5 (35.7) | 62 (42.8) | 4 (25.0) | 0.392⊛ |
| | Stage II | 2 (14.3) | 8 (5.5) | 1 (6.3) | |
| | Stage III | 0 (0.0) | 1 (0.7) | 0 (0.0) | |
| | Stage IV | 7 (50.0) | 74 (51.0) | 11 (68.8) | |
| Clinicopathological stage | Localized | 7 (50.0) | 70 (48.3) | 5 (31.3) | 0.463⊛ |
| | Locally advanced | 0 (0.0) | 1 (0.7) | 0 (0.0) | |
| | Advanced | 7 (50.0) | 74 (51.0) | 11 (68.8) | |
| Risk stratification | Low | 2 (14.3) | 13 (9.0) | 2 (12.5) | 0.998⊛ |
| | Intermediate | 4 (28.6) | 35 (24.1) | 5 (31.3) | |
| | High | 5 (35.7) | 66 (45.5) | 5 (31.3) | |
| | Very high | 3 (21.4) | 31 (21.4) | 4 (25.0) | |

#Divorced/Separated/Widowed *Fisher's exact p-value, ⊛Monte Carlo p-value

**Supplementary Table S4.** Multivariable regression results for socio-demographic and clinical covariates with no statistically significant associations across outcomes

| | High-risk disease | | Advanced disease | | High PSA at screening | |
|---|---|---|---|---|---|---|
| **Variable** | **aOR (95% CI)** | ***p-value*** | **aOR (95% CI)** | ***p-value*** | **aOR (95% CI)** | ***p-value*** |
| Marital status | | | | | | |
| Married | 2.6 (0.7–9.9) | 0.152 | 1.2 (0.3–4.3) | 0.790 | 2.9 (0.8–10.8) | 0.110 |
| Divorced/Separated/Widowed | 1.3 (0.3–6.7) | 0.757 | 2.2 (0.4–11.4) | 0.349 | 0.9 (0.2–4.6) | 0.907 |
| Single | Reference | — | Reference | — | Reference | — |
| Employment status | | | | | | |
| Unemployed/Retired | 1.0 (0.3–3.7) | 0.978 | 0.7 (0.2–2.2) | 0.549 | 1.0 (0.3–3.4) | 0.985 |
| Employed/Self-employed | Reference | — | Reference | — | Reference | — |
| Place of residence | | | | | | |
| Urban | 1.1 (0.5–2.5) | 0.821 | 1.3 (0.6–2.7) | 0.564 | 0.9 (0.4–2.0) | 0.727 |
| Peri-urban | 1.7 (0.5–5.5) | 0.361 | 0.8 (0.3–2.1) | 0.619 | 2.0 (0.6–6.5) | 0.239 |
| Rural | Reference | — | Reference | — | Reference | — |
